# Influence of DNA Type on the Physicochemical and Biological Properties of Polyplexes Based on Star Polymers Bearing Different Amino Functionalities

**DOI:** 10.3390/polym15040894

**Published:** 2023-02-10

**Authors:** Emi Haladjova, Silvia Panseri, Monica Montesi, Arianna Rossi, Athanasios Skandalis, Stergios Pispas, Stanislav Rangelov

**Affiliations:** 1 Institute of Polymers, Bulgarian Academy of Sciences, Acad. G. Bonchev st. bl.103A, 1113 Sofia, Bulgaria; 2 Institute of Science, Technology and Sustainability for Ceramics, National Research Council of Italy, Via Granarolo 64, 48018 Faenza, Italy; 3 Department of Chemical, Biological, Pharmaceutical and Environmental Sciences, University of Messina, 98166 Messina, Italy; 4 Theoretical and Physical Chemistry Institute, National Hellenic Research Foundation, 48 Vass. Constantinou Ave., 116 35 Athens, Greece

**Keywords:** gene delivery, star-shaped polymers, amino functionality, DNA structure, polycations, polyplexes, transfection

## Abstract

The interactions of two star polymers based on poly (2-(dimethylamino)ethyl methacrylate) with different types of nucleic acids are investigated. The star polymers differ only in their functionality to bear protonable amino or permanently charged quaternary ammonium groups, while DNAs of different molar masses, lengths and topologies are used. The main physicochemical parameters of the resulting polyplexes are determined. The influence of the polymer’ functionality and length and topology of the DNA on the structure and properties of the polyelectrolyte complexes is established. The quaternized polymer is characterized by a high binding affinity to DNA and formed strongly positively charged, compact and tight polyplexes. The parent, non-quaternized polymer exhibits an enhanced buffering capacity and weakened polymer/DNA interactions, particularly upon the addition of NaCl, resulting in the formation of less compact and tight polyplexes. The cytotoxic evaluation of the systems indicates that they are sparing with respect to the cell lines studied including osteosarcoma, osteoblast and human adipose-derived mesenchymal stem cells and exhibit good biocompatibility. Transfection experiments reveal that the non-quaternized polymer is effective at transferring DNA into cells, which is attributed to its high buffering capacity, facilitating the endo-lysosomal escape of the polyplex, the loose structure of the latter one and weakened polymer/DNA interactions, benefitting the DNA release.

## 1. Introduction

The delivery of nucleic acids is of great importance since it enables the development of modern therapeutic drugs and treatment strategies [1,2,3,4]. Nucleic acid transfer, however, is related to the use of vectors as vehicles to deliver the genetic material into the cells. Although approved therapies with viral vectors already exist [5], preference is currently given to non-viral carriers [6,7,8], since they display significant advantages and potential benefits including a controllable chemical diversity, possibilities for low costs, large-scale production in a more facile way, a larger payload capacity, better safety profiles and a reduced immunogenic response.

Cationic polymers are a major class of non-viral vectors for nucleic acids delivery [9,10,11]. Various polymer-based systems have been developed and studied as potential DNA carriers, though they have some limitations, which hinder their practical use [9,10,11]. The potential of cationic polymers containing amino or ammonium functionality is due to their ability to bind and condense negatively charged nucleic acids through electrostatic interactions. In the resulting nanoparticulate structures, known as polyplexes, nucleic acids molecules are compacted and protected from enzymatic degradation. Such compact structures facilitate the uptake, but for efficient delivery, a successful release of the nucleic acids’ cargo molecules is required. The polymer complexation ability is known to strongly depend on the amino functionality [7,9,11,12]. For example, quaternary ammonium groups and primary amine groups, most of which are protonated at the physiological pH, are permanently charged and characterized by a high binding affinity [13,14]. However, the nucleic acid release from the polyplexes is impeded by strong polymer/nucleic acid interactions. Polymers bearing secondary and tertiary amine groups form less strong complexes, but since these amine groups can be protonated, depending on pH of the medium, they are responsible for the successful endosomal escape of the polyplex and more efficient transfection [15,16]. The polymer/nucleic acid interactions are also known to depend on the molar mass of the two partners [17,18,19,20,21,22]. It has repeatedly been shown that the longer the polymer chain is, the stronger the nucleic acid binding affinity is [17,18,19,20]. The impact of the nucleic acid strands scale is significant as well [18,20,21,22]. Short linear DNA fragments (below 100 bp) are rigid and unable to bend spontaneously, whereas the longer molecules are flexible and capable of being shrunk and wrapped [23,24].

A number of physicochemical parameters of the resulting polyplexes, e.g., size, surface potential, colloidal stability in biological fluids, etc., are determinant factors for the biological performance of the systems [25]. However, a remaining challenge for the clinical utilization of such systems is the inherent cytotoxicity of the polyplexes due to the cationic character of the polymers utilized. Therefore, achieving a proper balance between a high transfection efficiency and a low toxicity profile is of significant importance. This relationship is known to strongly depend not only on the amino functionality (primary amino and quaternary ammonium groups are usually associated with high toxicity), but also on factors such as polymer composition, structure and topology, charge density, etc. [9,12,26]. Thus, polymers of branched topology were found to be more effective than the linear ones are, but they were also more toxic [27,28]. The molar mass is also known to strongly affect the efficiency/toxicity ratio, as high molar mass vectors are more effective, but they are associated with enhanced toxicity [26,29]. The type (RNA or DNA), structure (single or double stranded) and topology (linear or circular) of nucleic acids are also determinants of the behavior and physicochemical properties of the resulting vector systems [20,21,22].

We have previously evaluated and compared the physicochemical characteristics and biological performance of polyplexes based on linear copolymers composed of cationic blocks bearing tertiary amino and quaternary ammonium groups [30]. With the present study, we expand this work to investigate polymers of non-linear chain architecture, which, in general, exhibit a superior gene delivery ability, and to study the effects of the nucleic acid length and type. The aim of this work is to evaluate the interactions of two identical star-shaped polymers bearing tertiary amino or quaternary ammonium groups with nucleic acids of different molar mass and structure and to estimate how this relates to the physicochemical and biological behavior of the resulting systems. The polymers are based on poly(2-(dimethylamino)ethyl methacrylate) (PDMAEMA)—a highly promising cationic polymer with many desirable properties for developing efficient non-viral gene delivery systems. Polyplexes in a wide range of N/P ratios (N/P is the ratio of the positively charged polymer amine groups to negatively charged nucleic acid phosphate groups) were prepared using different types of nucleic acids: short linear segments, long linear molecules and circular/plasmid DNA. The effects of the length and topology of DNA and polymer amino functionality on the structure and properties of the resulting polyelectrolyte complexes were investigated by dynamic and electrophoretic light scattering. The cell viability and morphology, as well as in vitro transfection efficiency, of the investigated systems were evaluated using several representative human cell lines including osteosarcoma, osteoblast and human adipose-derived mesenchymal stem cells.

## 2. Materials and Methods

### 2.1. Materials

All of Sigma-Aldrich reagents were purchased from Merck-Bulgaria (Sofia, Bulgaria). Linear DNA sodium salt from salmon sperm (molar mass ~ 115 bp, denoted as lDNA 115) was purchased from Acros, linear DNA sodium salt from salmon testes (molar mass~2000 bp, denoted as lDNA 2000) was purchased from Sigma-Aldrich and plasmid DNA containing the gene encoding for the enhanced green fluorescent reporter protein (eGFP, molar mass~4730 bp, denoted as pDNA 4730) was purchased from Clontech (Palo Alto, CA, USA) and propagated in E. coli DH5 alpha strain (ThermoFisher Scientific, Walthman, MA, USA). For the preparation of DNA and polymer solutions, ultra-pure milliQ water (resistivity > 18 MΩ.cm) was used.

#### 2.1.1. Synthesis and Quaternization of Star-Like PDMAEMA

The synthesis of star-like poly(2-(dimethylamino)ethyl methacrylate) (PDMAEMA) was achieved by RAFT polymerization using the “arm-first” method. At first, low molecular weight, linear PDMAEMA arms [31] were prepared by RAFT polymerization using 2,2′-azobis(2-methylpropionitrile) (AIBN) and 4-cyano-4-((dodecylsulfanylthiocarbonyl)sulfanyl)pentanoic acid (CDTP) as the radical initiator and the chain transfer agent (CTA), respectively. The linear PDMAEMA homopolymers were used as macro-CTA for the synthesis of star-like PDMAEMA, with ethylene glycol dimethacrylate (EGDM) being the cross-linked core. The synthetic procedure has been described in detail elsewhere [32].

To impart permanent positive charges, the tertiary amine groups of the PDMAEMA arms were converted into quaternary ammonium groups via a quaternization reaction using methyl iodide (CH_3_I). The conversion was analyzed quantitatively, and more details about this procedure can be found elsewhere [32].

#### 2.1.2. Preparation of Polymer Solutions

The star-like PDMAEMA polymer was placed in an aqueous medium, followed by the addition of a weak acid (0.1 M HCl) until the full dissolution of the polymer. The quaternized polymer was spontaneously dissolved in pure water. The pH of all of the polymer solutions was adjusted to 7 by adding 0.1 M NaOH. The concentration of the stock polymer solutions was 1000 µg.mL^−1^.

#### 2.1.3. Polyplex Formation

The polyplexes were formed by mixing the polymer (100 µg.mL^−1^) and DNA (100 µg.mL^−1^ for lDNAs and 50 µg.mL^−1^ for pDNA) aqueous solutions at ambient temperature during vortexing. The amounts of polymer and DNA solutions were selected to obtain amino-to-phosphate groups (N/P) ratios in the 0.5–10 range.

#### 2.1.4. Cell Culture

Human osteosarcoma cell line (MG63, ATCC CRL-1427, ATCC, Manassas, VA, USA), human osteoblast cell line (hFOBs, ATCC CRL-11372, ATCC) and human adipose-derived mesenchymal stem cells (MSCs, ATCC PCS-500-011, ATCC) were used to test the materials. In detail, MG63 was cultured in DMEM/F-12 with GlutaMAX (Gibco, Langley, OK, USA), 10% Fetal Bovine Serum (FBS, Gibco) and 1% penicillin-streptomycin (pen/strep, 100 U.mL^−1^–100 µg.mL^−1^); hFOBs were cultured in DMEM/F-12 without phenol red (Gibco), with 10% Fetal Bovine Serum (FBS, Gibco) and 0.3 mg.mL^−1^ Geneticin (G418, Gibco); MSCs were cultured in Alpha-MEM Glutamax (Gibco), 15% Fetal Bovine Serum (FBS, Gibco), 10 ng.mL^−1^ recombinant human FGF-basic (Gibco) and 1% penicillin-streptomycin (pen/strep, 100 U.mL^−1^–100 µg.mL^−1^). The cell cultures were kept at 37 °C in an atmosphere of 5% CO_2_. The cells were detached from culture flasks by trypsinization, centrifuged and re-suspended in fresh media. The cell number and viability were assessed using a trypan blue dye exclusion test; all of the cell handling procedures were performed in a sterile laminar flow hood.

### 2.2. Methods

#### 2.2.1. Dynamic Light Scattering (DLS) and Electrophoretic Light Scattering (ELS)

The dynamic and electrophoretic light scattering measurements were performed on a 90Plus PALS instrument (Brookhaven Instruments Corporation) equipped with a 35 mW red diode laser (λ = 640 nm). The hydrodynamic diameter (D_h_) was determined at a scattering angle (θ) of 90°, while the ζ potential was determined by phase analysis light scattering (PALS) method at a scattering angle (θ) of 15°. Each measurement was performed in triplicate.

#### 2.2.2. Transmission Electron Microscopy (TEM)

The samples were examined using an high resolution TEM JEOL JEM-2100 electron microscope (JEOL, Tokyo, Japan) operating at 200 kV. They were prepared by depositing a drop of the solution onto a carbon grid.

#### 2.2.3. Buffering Capacity

The buffering capacity of the PDMAEMA stars was determined by standard acid-base titration. Polymer aqueous solutions (c = 500 µg.mL^−1^, V = 3 mL) were prepared, and the pH was adjusted to 10 by using 0.1 M NaOH. Then, titration with 0.1 M HCl to pH 3 was performed as 5 μL aliquots were added, and the pH was measured after each addition. Pure water was titrated as the control.

#### 2.2.4. Stability of Polyplexes in Presence of Salt

To preformed polyplexes dispersions at N/P = 8, increasing amounts of NaCl solutions in the range from 0.01 to 0.25 M were added. The stability of the complexes was monitored by dynamic light scattering measurements.

#### 2.2.5. Cell Viability Assay

Metabolically active cells were evaluated to assess the cell viability by performing an MTT assay. MSCs, hFOBs and MG63 cells were seeded in a 96-well plate (3.0 × 10^3^ cells/well). After 24 h, polyplexes dispersions prepared with lDNAs at N/P = 8 and diluted to different concentrations (5 µg.mL^−1^, 10 µg.mL^−1^, 20 µg.mL^−1^, 25 µg.mL^−1^, 40 µg.mL^−1^ and 50 µg.mL^−1^) were added to the wells. Additional experiments were performed with hFOBs and MG63 cells in contact with the two star-like polymers complexed with the eGFP plasmid for 24 h. For the cell viability analysis, the cell culture in contact with the polplexes was analyzed after 24 h, 48 h and 72 h. In detail, the reagent 3-(4,5-dimethylthiazol-2-yl)-2,5-diphenyltetrazolium bromide (MTT) was dissolved in PBS 1X (5 mg.mL^−1^) and incubated 1:10 for 2 h at 37 °C and 5% CO_2_ at each time point. The medium was removed and dimethyl sulfoxide (DMSO) was added to dissolve formazan crystals produced by the metabolically active cells. After 15 min, the absorbance was read at λ_max_ 570 nm using the Multiskan FC Microplate Photometer (ThermoFisher Scientific, Walthman, MA, USA), and it is proportionally related to the number of metabolically active cells. For each time point, three samples were analyzed.

#### 2.2.6. Cell Morphology Analysis

In a 24-well plate, 9.0 × 10^3^ cells/well (hFOBs, MG63 and MSCs) were seeded, and the day after, 20 µg.mL^−1^ of polyplexes dispersions prepared at N/P = 8 were added. The cell morphology was evaluated by observing the actin filaments after 24 h. The cells were washed two times with PBS 1X and fixed using 4% paraformaldehyde (Sigma) for 15 min at room temperature. Then, the fixed cells were permeabilized in PBS 1X with 0.1% (*v*/*v*) Triton X-100 (Sigma) for 5 min at room temperature. A PBS 1X wash was performed, and f-actin filaments were stained with FITC-conjugated fluorescein-phalloidin (LifeTechonologies, Carlsbad, CA, USA), followed by DAPI (600 nM, Invitrogen, Carlsbad, CA) counterstaining to identify the cell nuclei, following the manufacturer’s instructions. The samples were visualized using an inverted Ti-E Fluorescence Microscope (Nikon Corporation, Tokyo, Japan).

#### 2.2.7. In Vitro Transfection

In a 96-well plate, 1.0 × 10^4^ cells/well (hFOBs and MG63) were seeded. The following day, the media were removed, and the Opti-MEM Reduced Serum Medium (Gibco) with polyplex dispersions prepared with pDNA at N/P = 8 were added. The cells were transfected with 800 ng plasmid DNA. The polyplex dispersions were used at the final concentration of 20 μg.mL^−1^. Lipofectamine 2000 Transfection Reagent (Invitrogen) was used as control. After 24 h, the transfection solution was removed and replaced with completed culture media. After 48 h, the cells were fixed and permeabilized as described above, then washed with PBS 1X, and DAPI (Invitrogen) staining was performed to reveal the cell nuclei. Fluorescence was derived from the transfection, and DAPI stains were detected, and images were acquired by using an Inverted Ti-E Fluorescent Microscope (Nikon Corporation, Tokyo, Japan). The transfection efficiency (% respect to Lipofectamine 2000) was calculated by counting the green fluorescence cells on the total cells number in a field; 8 fields for each condition were analyzed.

#### 2.2.8. Statistical Analysis

A two-way analysis of variance (ANOVA) test was performed to elaborate the results of the MTT assay, and they were analyzed by using Tukey’s multiple comparisons test as a post hoc test. The results are expressed as mean ± standard error of the mean (SEM) plotted on the graph. Statistical analyses were performed by GraphPad Prism software (version 8.0.1, GraphPad Software, San Diego, CA, USA).

## 3. Results and Discussion

### 3.1. Characterization and Aqueous Solution Properties of the Star-Shaped Polymers

**Figure 1:**
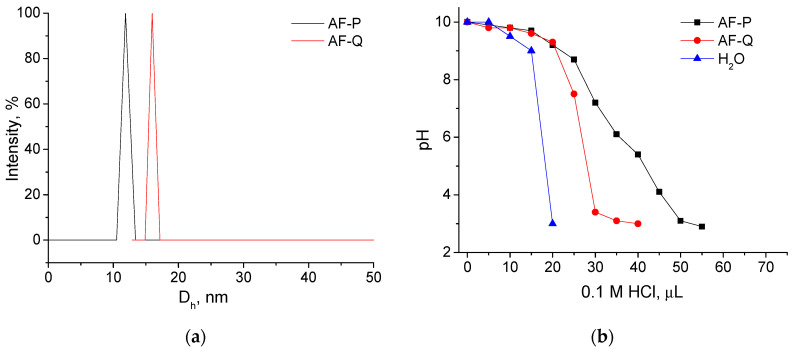
Size distribution curves from DLS of star-shaped PDMAEMA polymers in water at 25 °C and 100 μg.mL^−1^ (**a**). pH versus volume of 0.1 M HCl curves for star-shaped PDMAEMA polymers (**b**). The titration was performed at 25 °C and initial concentration and volume of 500 μg.mL^−1^ and 3 mL, respectively.

The star-like PDMAEMA (hereinafter AF-P) was synthesized via RAFT polymerization using the “arm-first” method, which has been described in detail elsewhere [32]. The polymer consists of a cross-linked core to which an average of 25 PDMAEMA arms are attached; the average degree of polymerization of each arm is 23 [32]. The resulting molar mass (Table 1) is within the range of the molar masses of PDMAEMA-based polymers that is deemed to be optimal to achieve a decent transfection efficiency/toxicity ratio [26]. PDMAEMA polymers with larger molar mass exhibited an increased transfection efficiency, but at the same time, high toxicity [26]. In a wide range of concentration (100–1000 μg.mL^−1^) in an aqueous solution, the AF-P polymer is characterized by a constant size and ζ potential (Table 1). AF-P exhibited strongly positive ζ-potential due to the partial protonation state of the tertiary amino groups in water [33,34]. The size distribution from DLS was narrow (Figure 1a, black curve), with a mean hydrodynamic diameter (D_h_) of 11.4 nm. The later suggested molecularly dissolved state of the star-shaped polymer in the investigated concentration range.

**Scheme 1:**
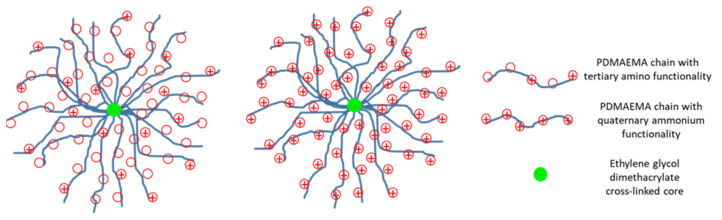
Schematic presentation of the structure of the PDMAEMA-based star-like polymers.

The tertiary amines of AF-P polymer were converted into quaternary ammonium groups in order to impart permanent positive charges. This was achieved by a typical quaternization reaction using methyl iodide [32]. Thus, two identical star-like polymers bearing tertiary amino or quaternary ammonium groups and differing in their total molar masses (Table 1) were obtained. Their structure is schematically presented in Scheme 1. As expected, the quaternized polymer (hereafter, AF-Q) exhibited more positive ζ-potential than the parent AF-P polymer did (Table 1). The D_h_ of the AF-Q stars was slightly higher (15.8 nm), which is probably due to the presence of iodine counterion and the stronger electrostatic repulsion of the quaternized polymer chains. It is of note that the narrow size distribution was preserved, as evident from Figure 1a. The value of D_h_ suggested that AF-Q was also molecularly dissolved in the investigated concentration range.

Standard acid-base titration over a pH range from ten to three was employed to evaluate the buffering capacity of the two star-shaped polymers. The buffering capacity and the proton sponge effect are considered to be responsible for the endo-lysosomal escape of polyplexes, which is of paramount importance to avoid the enzymatic degradation of the polyplexes within the lysosomal compartments. The titration curves are presented in Figure 1b; pure water was titrated as a control. As is evident, the AF-P polymer is characterized by a gradual change in the pH during titration due to the ability of protonable tertiary amino groups to trap protons. In contrast, the change in the pH for the quaternized AF-Q polymer was abrupt, owing to the lack of protonable moieties. In addition, an intrinsic buffering capability was calculated using the reciprocal value of the slope of curves, and the values are given in Table 2. The higher buffering capacity of the parent AF-P polymer revealed its potential to facilitate the endo-lysosomal escape.

### 3.2. Interaction/Complexation of PDMAEMA Stars with Nucleic Acids

The interactions of the star-shaped PDMAEMA polymers with nucleic acids of different molar masses and structures were investigated by DLS, ELS and TEM. Polyplexes in a wide range of N/P ratios were prepared using short linear segments (115 bp, hereinafter, lDNA 115), long linear molecules (2000 bp, hereinafter, lDNA 2000) and circular/plasmid DNA (4730 bp, hereinafter, pDNA 4730). All of the complexes were spontaneously formed in water at room temperature.

**Figure 2:**
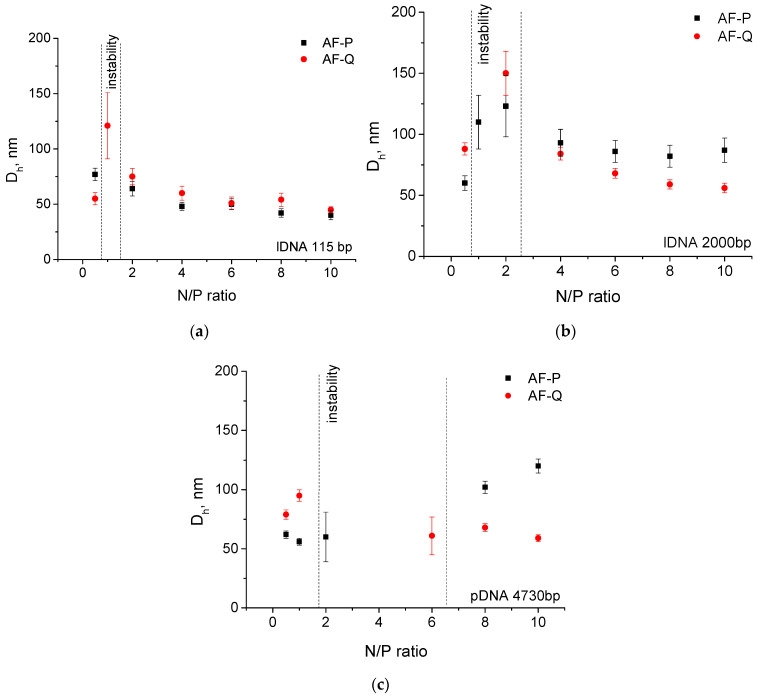
Variations of the hydrodynamic diameter, D_h_, with the N/P ratio of polyplexes based on PDMAEMA star-shaped polymers prepared with (**a**) lDNA 115, (**b**) lDNA 2000 and (**c**) pDNA 4730.

The variations in the hydrodynamic diameter with the N/P ratio are shown in Figure 2. Areas of instability were entered at certain N/P ratios, in which strong data scattering, low reproducibility of results and occasional formation of very large particles and/or precipitates were detected. The instability areas, which are observable in the same N/P intervals for AF-P and AF-Q, gradually broadened and shifted to higher N/P ratios with increasing molar mass of DNA (Figure 2). Due to the star-like topology of the polymers, some of the amino or ammonium groups of the polymers were inaccessible, particularly for longer DNA molecules (lDNA 2000) and/or more rigid structure of the plasmid DNA (pDNA 4730) [22,35,36], which required larger amounts of polymer for neutralization and, hence, broadening and shifting of the instability areas to higher N/P ratios.

Outside the instability area, colloidally stable, narrowly distributed (PDI in the 0.075–0.105 range) and relatively small polyplex particles are observed (50–125 nm, Figure 2). Compared to the size of a single star-like macromolecule (Figure 1a, Table 1), they are considerably larger, implying that the polyplex particles consist of several polymer molecules. Beyond the instability areas, that is, in the excess of polymers, the size of the polyplex particles tends to increase with increasing DNA molar mass. Thus, the size of the polyplex particles that lDNA 115 formed with AF-P and AF-Q polymers was around 50 nm (Figure 2a). It was in the range from 50 to 85 nm for the polyplexes with lDNA 2000 (Figure 2b) to reach 50–125 nm for those with pDNA 4730 (Figure 2c).

The effects of tertiary amino vs. quaternary ammonium functionality are expressed in terms of the decreasing size of the polyplexes formed by AF-Q. This was pronounced for lDNA 2000 and pDNA 4730 (Figure 2b,c), whereas for the shortest DNA, the differences were insignificant (Figure 2a). The smaller sizes of the polyplexes that AF-Q formed may indicate the stronger binding affinity of the polymer bearing quaternary ammonium functionality and the formation of more compact and tighter particles, as previously observed [30,37,38].

**Figure 3:**
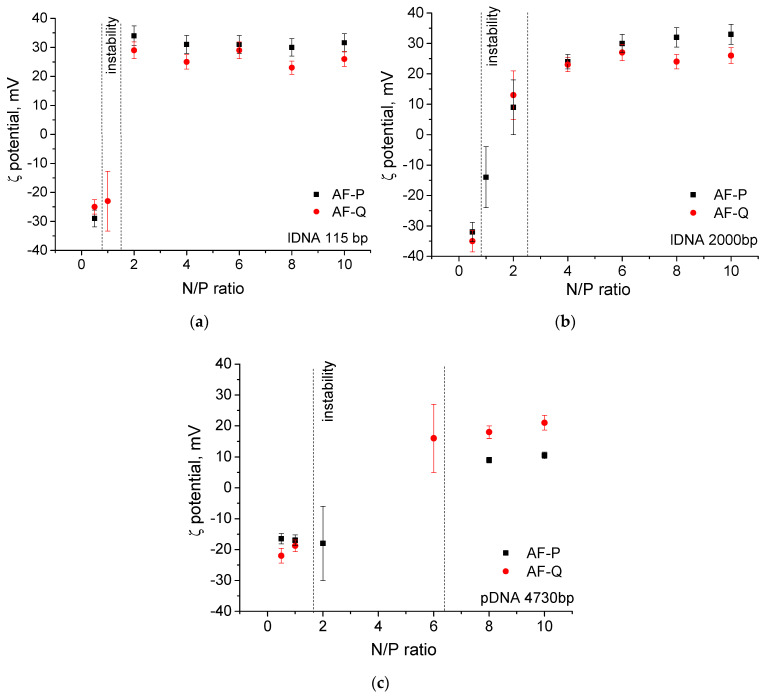
Variations of the ζ potential with the N/P ratio of polyplexes based on PDMAEMA star-shaped polymers prepared with (**a**) lDNA 115, (**b**) lDNA 2000 and (**c**) pDNA 4730.

The variations in the ζ potential of the polyplexes were also informative of the complexation of the investigated star-shaped polymers with DNA. A typical sigmoidal curve pattern with a transition from negative to positive values of the ζ potential with an increasing N/P ratio was identified in all of the polymer-to-nucleic acid formulations (Figure 3). Similarly to the instability areas of D_h_ variations, the transition intervals transformed from sharp and sudden to more gradual ones, whereas their positions shifted to higher N/P ratios with the molar mass (bp) and type (linear and plasmid) of DNA (cf. Figure 2 and Figure 3). Besides the position and width of the transition intervals, the magnitude of the ζ potential beyond the transition was also influenced by the molar mass and type of DNA, being higher for the polyplexes of lDNA 115, which is most probably due to the easier access of its small molecules to the amino or ammonium groups of the star-like polymers than that of the larger molecules (lDNA 2000) and the more rigid plasmid structure (pDNA 4730). The effects of the polymer functionality (amino vs. ammonium) were more pronounced for the polyplexes with the plasmid DNA; beyond the transition, the AF-Q/pDNA 4730 complexes exhibited a more positive ζ potential than those of AF-P (Figure 3c). Referring to the smaller size of the former one (Figure 2c), the stronger complexation ability and the formation of more dense and tight complexes could be associated with the polymer bearing strongly positively charged quaternary ammonium groups.

**Figure 4:**
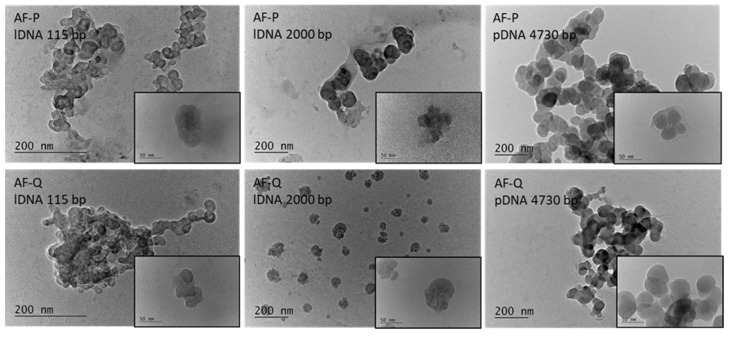
TEM micrographs of polyplexes based on PDMAEMA star–shaped polymers prepared at N/P ratio of 8. Scale bars of the insets 50 nm.

The morphologies of the polyplexes were visualized by TEM. Representative micrographs are shown in Figure 4. An abundance of clusters composed of mostly spherical, individual particles was observed. The dimensions of the individual particles (inset pictures in Figure 4) were in good agreement with the results from the DLS, implying that the large clusters had been formed during the preparation of the samples for observation. The polyplex particles formed by the linear DNAs (lDNA 115 and lDNA 2000) were of a shape that somewhat deviates from sphericity. In addition, these objects were of high and homogeneous electron density, looking similar to coiled threads, inferring that the linear DNAs were probably condensed by the star-shaped polymers into compact and tight polyplex particles, as suggested above. In some aspects, the morphologies of the polyplexes formed with pDNA 4730 were different. An interesting finding is depicted in the inset. It is composed of four individual spherical objects with dimensions that roughly correspond to the dimensions of the star-shaped polymers from Table 1. Supposedly, the whole structure is held together by the plasmid DNA molecules wrapping the individual polymer spheres. Interestingly, objects such as this were observed also for the formulations with lDNA 2000 (inset), but less frequently, and never for those with the shortest DNA.

### 3.3. Salt-Induced Destabilization of Polyplexes

**Figure 5:**
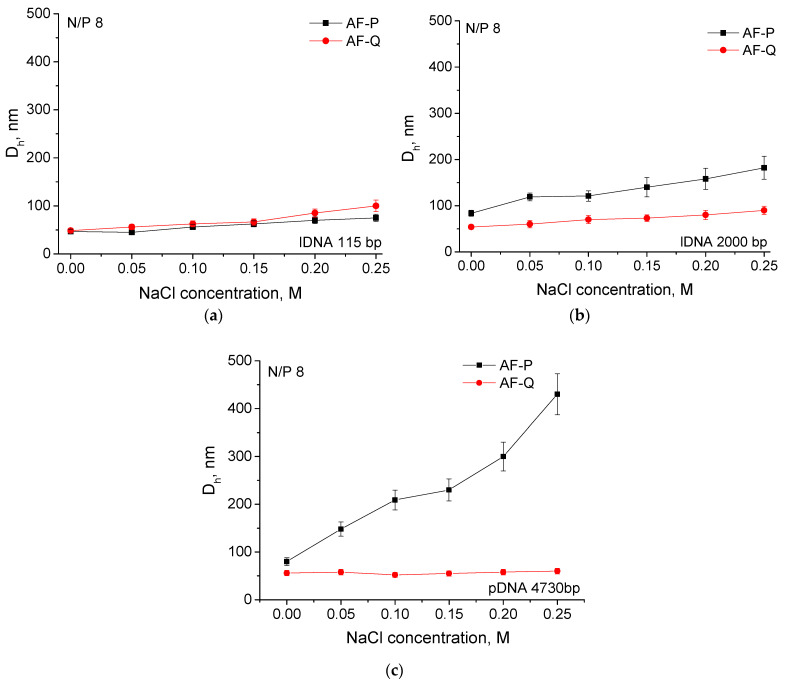
Variations of the hydrodynamic diameters, D_h_, with NaCl concentration of polyplexes based on PDMAEMA star-shaped polymers prepared at N/P ratio of 8 with (**a**) lDNA 115, (**b**) lDNA 2000 and (**c**) pDNA 4730.

The release of DNA from the polyplexes’ structures is a fundamental prerequisite for the efficiency of the systems. It is known that the addition of salt causes charge screening and may result in the weakening of the electrostatic interactions between the polymer and DNA phosphate groups, causing the destabilization of the complexes [30,37,39]. Furthermore, the interactions of a nucleic acid delivery system with biological fluids are ubiquitous in the delivery process. Therefore, the stability of the investigated polyplexes upon the addition of increasing amounts of salt to preformed polyplexes was investigated. N/P = 8 was selected for this study, as well as for the investigation of the biological performance of the polyplexes. As discussed in the previous section, N/P = 8 was positioned outside the instability areas, where a high reproducibility of results in terms of physiscochemical characteristics and properties of the polyplexes was exhibited. The further increase in the N/P ratio may negatively affect the biological tolerance of the polyplexes. The size variations were monitored by DLS (Figure 5). It is observable from Figure 5 that all of the polyplexes exhibited a gradual increase in size, indicating sensitivity to the addition of salt. The size increase is relatively small (up to 100 %) for the polyplexes of the quaternized polymer AF-Q with all the three types of DNA, as well as for the AF-P/lDNA 115, and it can be attributed to the loosening of the complexes resulting from the weakening of the electrostatic interactions and their swelling due to the insertion of water molecules in their structures. The size increase was considerably larger for the complexes of AF-P with the longer DNAs—lDNA 2000 and pDNA 4730—and revealed the impact of the DNA length. Particularly for the polyplex with the plasmid DNA, it reached 440%, which was too large to be attributed solely to swelling of the polyplex. The following scenario, which is supported by results from a previous study [30,40], is suggested: upon loosening of the polyplex structure, the segments of the long DNA chains can be partially dissociated, and thus, are free to participate in complexation with other polyplexes. Thus, large superaggregates of loose polyplexes were formed.

### 3.4. Biological Performance of Polyplexes

#### 3.4.1. Cytotoxicity of Polyplexes Formed with lDNA

**Figure 6:**
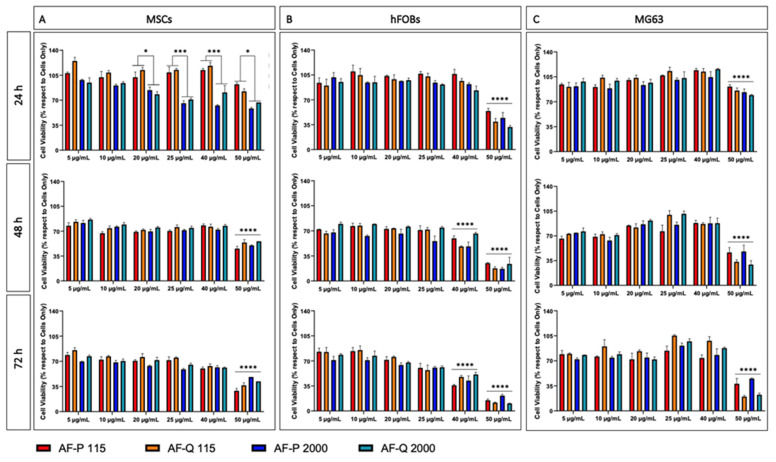
Cell viability analysis. MTT assay was performed after 24 h, 48 h and 72 h of being in the culture. MSCs (**A**), hFOBs (**B**) and MG63 (**C**) were cultured in contact with 5 concentrations of AF-P/lDNA 115, AF-Q/lDNA 115, AF-P/lDNA 2000 and AF-Q/lDNA 2000 (5 µg.mL^−1^; 10 µg.mL^−1^; 20 µg.mL^−1^; 25 µg.mL^−1^; 40 µg.mL^−1^; 50 µg.mL^−1^) at N/P of 8. The data show the percentage of viable cells as compared to cells only as the control, and the mean ± SEM are presented. * *p* value < 0.05, *** *p* value < 0.001, **** *p* value < 0.0001.

A detailed cytotoxic study of polyplexes formed with PDMAEMA star-shaped polymers and linear DNA was performed. Cell viability and morphology were evaluated after the cells were exposed to polyplexes formed from both AF-P and AF-Q polymers and lDNA 115 or lDNA 2000 at an N/P ratio of 8 to determine the cytotoxic effects. In detail, we checked the cell viability and proliferation over time of three human cell types in contact with several polyplex concentrations (5 μg.mL^−1^, 10 μg.mL^−1^, 20 μg.mL^−1^, 25 μg.mL^−1^, 40 μg.mL^−1^ and 50 μg.mL^−1^). MG63, MSCs and hFOBs cells were selected as potential human cell targets for the development of new therapies based on nucleic acid delivery for the treatment of different pathologies (e.g., cancer) or to enhance tissue regeneration. The results showed a cell viability reduction after 24 h in the MSCs due to the presence of AF-P/lDNA 2000 and AF-Q/lDNA 2000 particles with respect to AF-P/lDNA 115 and AF-Q/lDNA 115, respectively, with a statistically significant difference starting from 20 µg.mL^−1^ (Figure 6A). This initial difference disappeared after 48h and 72h, where only a significant decrease in cell viability is visible with the higher concentration of all of the polyplex dispersions (i.e., 50 µg.mL^−1^) compared to the other concentrations, where the cells viability is about 80–70% with respect to the cells only (Figure 6A).

A different cell behavior was detected with the hFOBs. In fact, after 24 h, there were no significant differences among the groups, and all of the systems based on AF-P and AF-Q polymers with lDNA 115 and lDNA 2000 showed a cell viability that was comparable to the cells only group, except for the higher polyplex concentrations (Figure 6B). Over time and by increasing the polyplexes concentration, a remarkable reduction of cell viability without significant differences among the groups was observed, especially with the 40 and 50 µg.mL^−1^ concentrations.

The MG63 cells were less sensitive to the presence of all of the polyplexes (Figure 6C). After 24 h, the cell viability was over 75–80% with respect to the cells only for all the concentrations. Only starting from 48 h, the 50 µg.mL^−1^ polyplex solutions induced a strong cell viability reduction, but without any statistically significant differences among the groups (Figure 6C).

**Figure 7:**
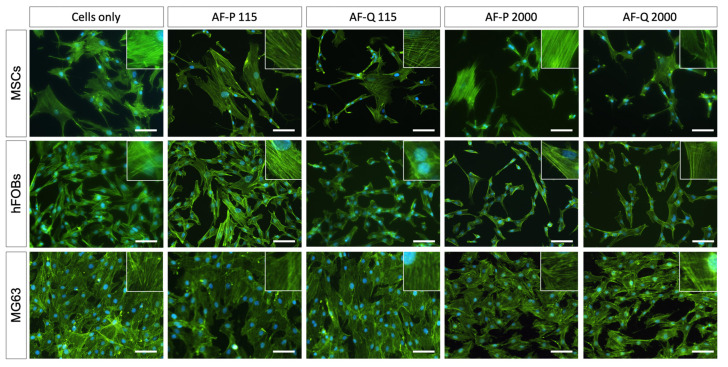
Morphological analysis of MSCs, hFOBs and MG63 cultured in presence of 20 µg.mL^−1^ of polyplexes at N/P of 8 for 24 h. Microspheres evaluated by phalloidin staining (green: actin filaments; blue: cell nuclei). Insets: details at higher magnification. Scale bars: 100 µm.

A qualitative cell morphology analysis was performed after 24 h with one concentration (20 µg.mL^−1^) (Figure 7). The organization of the cytoskeletal structure of actin filaments is an essential element in maintaining and modulating the cellular morphology and cell structural integrity. The detection of actin filaments confirmed the cell viability data. In detail, a lower MSCs density was detected with AF-P/lDNA 2000 and AF-Q/lDNA 2000 compared to those of AF-P/lDNA 115 and AF-Q/lDNA 115. In addition, even the MSCs’ morphologies seemed to be compromised when the cells were cultured in the presence of AF-P/lDNA 2000 and AF-Q/lDNA 2000. In contrast, the polyplexes did not compromise the cytoplasmatic morphology in hFOBs and MG63. The cells were well spread, showing the typical cytoplasmic elongations without the presence of morphological cell damage markers (Figure 7).

The observed deviation from the complexes formed with the AF-P and AF-Q polymers with lDNA 2000 could be due to their slightly larger size compared to those formed with lDNA 115. However, the cytotoxic evaluation of the systems revealed that they were sparing with respect to the cell lines studied. The polyplexes based on both the AF-P and AF-Q polymers exhibited good biocompatibility without disrupting the cytoskeletal structure of the actin filaments.

#### 3.4.2. Cytotoxicity and Transfection Efficiency of Polyplexes Formed with pDNA

**Figure 8:**
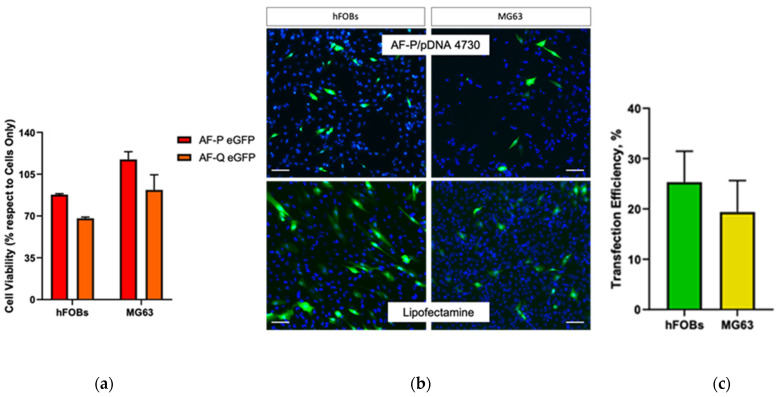
(**a**) Cells viability analysis. MTT assay was performed after 24 h of culture. hFOBs and MG63 were cultured in contact with 20 µg.mL^−1^ of AF-P eGFP and AF-Q eGFP at N/P of 8. The data show the percentage of viable cells as compared to cells only, as the control and the mean ± SEM are presented. (**b**) Qualitative transfection efficacy images. eGFP positive cells in green and cell nuclei in blue. Scale bars: 100 µm. (**c**) Transfection efficiency. % of transfection efficiency of AF-P eGFP complex at N/P of 8 compared to Lipofectamine used as control group.

The cytotoxicity test of the polyplexes formed with pDNA 4730 was performed with the hFOBs and MG63 cell lines at an N/P ratio of 8. A single concentration of 20 µg.mL^−1^ was tested. AF-P/pDNA 4730 did not negatively affect the cell viability of both of the cell types after 24 h, whereas AF-Q/pDNA 4730 slightly decreased the cell viability (Figure 8a).

The ability of AF-P and AF-Q polymers to transfer pDNA 4730 into the cells was investigated as well. The transfection efficiency was analysed by the quantification of the eGFP positive cells by counting the number of green transfected cells in the total cell number, as assessed by DAPI staining. The results showed that the AF-Q/pDNA 4730 complexes did not transfect the cells, in fact, no eGFP positive cells were detected. The lack of transfected cells with the AF-Q polymer is probably due to the formed dense complexes and the resulting inability to release DNA. In contrast, AF-P/pDNA 4730 complexes promoted plasmid DNA internalization and expression, with a good fluorescence intensity level being detected in both of the cell types (Figure 8b). The transfection efficiencies of AF-P/pDNA 4730 and Lipofectamine, which was used in the control group, in the hFOBs and MG63 cells were 25.3 ± 6.2% and 19.4 ± 6.1%, respectively (Figure 8c). The positive results observed with AF-P polymer could be related to both the looser structure of these polyplexes, enabling the release of DNA molecules and the presence of protonable tertiary amino groups, promoting the effective endo-lysosomal escape of the polyplex particles.

## 4. Conclusions

Two identical star-like polymers bearing tertiary amino or quaternary ammonium groups were used in this study. They were prepared by RAFT polymerization of 2-(dimethylamino)ethyl methacrylate) using the “arm-first” approach and further modified via quaternization of the tertiary amino groups. The two polymers were of the same chain architecture (star-like), number and polymerization degree of the arms, and they differed only in the functionality—tertiary amino vs. quaternary ammonium. The polymers were molecularly dissolved in water and were characterized with a relatively small size (11.4 nm, 15.8 nm) and strongly positive (24.1 mV, 31.6 mV) ζ potential. Whereas the quaternized polymer exhibited a slightly larger size and a more positive ζ potential, the parent polymer was characterized by a higher buffering capacity due to the ability of the tertiary amino groups to absorb protons. Both polymers spontaneously formed polyelectrolyte complexes with nucleic acids of different lengths and types in a wide range of N/P ratios. The effects of the DNA length/type were expressed in the position and width of the instability areas/transition intervals (being wider and shifted to higher N/P ratios for the longer and plasmid DNA), the size of the polyplex particles (tended to increase with increasing molar mass/length of DNA), and the magnitude of the ζ potential (more positive for the polyplexes of the shorter DNA). The addition of NaCl contributed to weakening of the electrostatic interactions and loosening of the polyplexes, which were strongly pronounced for the polyplexes of the parent AF-P polymer with the longer (lDNA 2000) and plasmid (pDNA 4730) DNA. In general, the size was smaller and the ζ potential was more positive for the polyplex particles of the quaternized AF-Q polymer, particularly with the plasmid DNA, compared to those of the parent AF-P polymer due to the stronger binding and formation of more compact and tighter polyplex particles. The cytotoxicity of the polyplexes at N/P = 8 was evaluated for three human cell types—the osteosarcoma cell line (MG63), the osteoblast cell line (hFOBs), and adipose-derived mesenchymal stem cells (MSCs). A remarkable reduction of the cell viability without significant differences among the groups was observed only at the highest polyplex concentrations (40 and/or 50 µg.mL^−1^, depending on the cell type). The transfection efficiencies of the polyplexes of the two polymers were examined in the hFOBs and MG63 cell lines. The loose structure of the AF-P polyplexes and the presumed abilities to promote effective endo-lysosmal escape and the release of DNA appeared to be favorable features for the enhanced effectiveness of the parent, non-quaternized PDMAEMA star-like polymer to transfect the cells.

## Figures and Tables

**Table 1:** Molecular and physicochemical characteristics of PDMAEMA star-shaped polymers.

Sample	Code	M_w_, ^(a)^ g/mol	M_w_/M_n_ ^(a)^	Number of Arms ^(b)^	DP of Arm ^(b)^	D_h_, ^(c)^ nm (PDI ^(e)^)	ζ, ^(d)^ mV
**Arm-first PDMAEMA**	AF-P	89 000	1.37	25	23	11.4 (0.091)	24.1
**Arm-first QPDMAEMA**	AF-Q	169 000	1.37	25	23	15.8 (0.083)	31.6

^(a)^ M_w_, M_n_ and M_w_/M_n_ are weight-averaged molar mass, number-averaged molar mass and molar mass distribution, respectively, from size exclusion chromatography [32]; ^(b) 1^H NMR [31]; ^(c)^ DLS; ^(d)^ ELS were performed in water at 25 °C and 100 μg.mL^−1^; ^(e)^ the values of polydispersity index (PDI) are given in parenthesis.

**Table 2:** Buffering capacity of PDMAEMA star-shaped polymers given as reciprocal value of the slope of curves from Figure 1b over a pH range from 10 to 3.

Sample	Buffering Capacity, 1/Slope,
AF-P	6.76
AF-Q	4.54
H_2_O	3.31

## Data Availability

Data is contained within the article.

## References

[1] Shi B., Zheng M., Tao W., Chung R., Jin D., Ghaffari D., Farokhzad O.C. (2017). Challenges in DNA delivery and recent advances in multifunctional polymeric DNA delivery system. Biomacromolecules.

[2] Zhang Y., Satterlee A., Huang L. (2012). In vivo gene delivery by nonviral vectors: Overcoming hurdles?. Mol. Ther..

[3] Ibraheem D., Elaissari A., Fessi H. (2014). Gene therapy and DNA delivery systems. Int. J. Pharm..

[4] Patil S.D., Rhodes D.G., Burgess D.J. (2005). DNA-based therapeutics and DNA delivery systems: A comprehensive review. AAPS J..

[5] Young L.S., Searle P.F., Onion D., Mautner V. (2006). Viral gene therapy strategies: From basic science to clinical application. J. Pathol..

[6] Hill A., Chen M., Chen C.-K., Pfeifer B.A., Jones C. (2016). Overcoming gene-delivery hurdles: Physiological considerations for nonviral vectors. Trends Biotechnol..

[7] Lai W.F., Wong W.T. (2018). Design of polymeric gene carriers for effective intracellular delivery. Trends Biotechnol..

[8] Yin H., Kanasty R.L., Eltoukhy A.A., Vegas A.J., Dorkin J.R., Anderson D.G. (2014). Non-viral vectors for gene-based therapy. Nat. Rev. Genet..

[9] Aied A., Greiser U., Pandit A., Wang W. (2013). Polymer gene delivery: Overcoming the obstacles. Drug Discov..

[10] O’Rorke S., Keeney M., Pandit A. (2010). Non-viral polyplexes: Scaffold mediated delivery for gene therapy. Prog. Polym. Sci..

[11] Eliyahu H., Barenholz Y., Domb A.J. (2005). Polymers for DNA delivery. Molecules.

[12] Jones C.H., Chen C.-K., Ravikrishnan A., Rane S., Pfeifer B.A. (2013). Overcoming nonviral gene delivery barriers: Perspective and future. Mol. Pharm..

[13] Izumrudov V.A., Zhiryakova M.V., Kudaibergenov S.E. (1999). Controllable stability of DNA-containing polyelectrolyte complexes in water-salt solutions. Biopolymers.

[14] Varkouhi A.K., Mountrichas G., Schiffelers R.M., Lammers T., Storm G., Pispas S., Hennink W.E. (2012). Polyplexes based on cationic polymers with strong nucleic acid binding properties. Eur. J. Pharm. Sci..

[15] Bus T., Traeger A., Schubert U.S. (2018). The great escape: How cationic polyplexes overcome the endosomal barrier. J. Mater. Chem. B.

[16] Warriner L.W., Duke III J.R., Pack D.W., DeRouchey J.E. (2018). Succinylated polyethylenimine derivatives greatly enhance polyplex serum stability and gene delivery in vitro. Biomacromolecules.

[17] Godbey W.T., Wu K.K., Mikos A.G. (1999). Size matters: Molecular weight affects the efficiency of poly(ethylenimine) as a gene delivery vehicle. J. Biomed. Mater. Res..

[18] Blakney A.K., Yilmaz G., McKay P.F., Becer C.R., Shattock R.J. (2018). One size does not fit all: The effect of chain length and charge density of poly(ethylene imine) based copolymers on delivery of pDNA, mRNA, and RepRNA polyplexes. Biomacromolecules.

[19] Schaffer D.V., Fidelman N.A., Dan N., Lauffenburger D.A. (2000). Vector unpacking as a potential barrier for receptor-mediated polyplex gene delivery. Biotechnol. Bioeng..

[20] Shen Z.L., Xia Y.Q., Yang Q.S., Tian W.D., Chen K., Ma Y.Q., Cheng Y. (2017). Polymer–Nucleic Acid Interactions In Polymeric Gene Delivery Systems.

[21] Jiang Y., Reineke T.M., Lodge T.P. (2018). Complexation of DNA with Cationic Copolymer Micelles: Effects of DNA Length and Topology. Macromolecules.

[22] Tan Z., Jiang Y., Zhang W., Karls L., Lodge T.P., Reineke T.M. (2019). Polycation Architecture and Assembly Direct Successful Gene Delivery: Micelleplexes Outperform Polyplexes via Optimal DNA Packaging. J. Am. Chem. Soc..

[23] Ugrinova I., Mitkova E., Moskalenko C., Pashev I., Pasheva E. (2007). DNA Bending versus DNA End Joining Activity of HMGB1 Protein Is Modulated in Vitro by Acetylation. Biochemistry.

[24] Kahn J.D. (2014). DNA, Flexibly Flexible. Biophys. J..

[25] Yue Y., Wu C. (2013). Progress and perspectives in developing polymeric vectors for in vitro gene delivery. Biomater. Sci..

[26] Agarwal S., Zhang Y., Maji S., Greiner A. (2012). PDMAEMA based gene delivery materials. Mater. Today.

[27] Dai F., Sun P., Liu Y., Liu W. (2010). Redox-cleavable star cationic PDMAEMA by arm-first approach of ATRP as a nonviral vector for gene delivery. Biomaterials.

[28] Mendrek B., Sieroń Ł., Libera M., Smet M., Trzebicka B., Sieroń A.L., Dworak A., Kowalczuk A. (2014). Polycationic star polymers with hyperbranched cores for gene delivery. Polymer.

[29] Eltoukhy A.A., Siegwart D.J., Alabi C.A., Rajan J.S., Langer R., Anderson D.G. (2012). Effect of molecular weight of amine end-modified poly(β-amino ester)s on gene delivery efficiency and toxicity. Biomaterials.

[30] Haladjova E., Chrysostomou V., Petrova M., Ugrinova I., Pispas S., Rangelov S. (2021). Physicochemical Properties and Biological Performance of Polymethacrylate based Gene Delivery Vector Systems: Influence of Amino Functionalities. Macromol. Biosci..

[31] Skandalis A., Pispas S. (2017). PDMAEMA-b-PLMA-b-POEGMA triblock terpolymers via RAFT polymerization and their self-assembly in aqueous solutions. Polym. Chem..

[32] Skandalis A., Pispas S. (2019). Synthesis of (AB)n-, AnBn-, and AxBy-type amphiphilic and double-hydrophilic star copolymers by RAFT polymerization. J. Polym. Sci. A Polym. Chem..

[33] van de Wetering P., Moret E.E., Schuurmans-Nieuwenbroek N.M., van Steenbergen M.J., Hennink W.E. (1999). Structure-activity relationships of water-soluble cationic methacrylate/methacrylamide polymers for nonviral gene delivery. Bioconjug. Chem..

[34] Lee H., Son S.H., Sharma R., Won Y.-Y. (2011). A discussion of the pH-dependent protonation behaviors of poly(2-(dimethylamino)ethyl methacrylate) (PDMAEMA) and poly(ethylenimine-ran-2-ethyl-2-oxazoline) (P(EI-r-EOz)). J. Phys. Chem. B.

[35] Dhanoya A., Chain B.M., Keshavarz-Moore E. (2011). The impact of DNA topology on polyplex uptake and transfection efficiency in mammalian cells. J. Biotechnol..

[36] Haladjova E., Halacheva S., Posheva V., Peycheva E., Moskova-Doumanova V., Topouzova-Hristova T., Doumanov J., Rangelov S. (2015). Comb-like Polyethyleneimine-based Polyplexes: Balancing Toxicity, Cell Internalization, and Transfection Efficiency via Polymer Chain Topology. Langmuir.

[37] Lee J.H., Lim Y.B., Choi J.S., Lee Y., Kim T.I., Kim H.J., Yoon J.K., Kim K., Park J.S. (2003). Polyplexes assembled with internally quaternized PAMAM-OH dendrimer and plasmid DNA have a neutral surface and gene delivery potency. Bioconjug. Chem..

[38] Vader P., van der Aa L.J., Engbersen J.F.J., Storm G., Schiffelers R.M. (2012). Physicochemical and Biological Evaluation of siRNA Polyplexes Based on PEGylated Poly(amido amine)s. Pharm. Res..

[39] Lai E., van Zanten J.H. (2001). Monitoring DNA/Poly-l-Lysine Polyplex Formation with Time-Resolved Multiangle Laser Light Scattering. Biophys. J..

[40] Chrysostomou V., Katifelis H., Gazouli M., Dimas K., Demetzos C., Pispas S. (2022). Hydrophilic random cationic copolymers as polyplex-formation vectors for DNA. Materials.

